# Greenness and whiteness assessment of a sustainable voltammetric method for difluprednate estimation in the presence of its alkaline degradation product

**DOI:** 10.1038/s41598-024-61712-0

**Published:** 2024-05-27

**Authors:** Heidi R. Abd El-Hadi, Maya S. Eissa, Basma M. Eltanany, Hala E. Zaazaa, Reham M. Arafa

**Affiliations:** 1 Faculty of Pharmacy, Pharmaceutical Chemistry Department, Egyptian Russian University, Badr City, Cairo, Egypt; 2 Faculty of Pharmacy, Analytical Chemistry Department, Cairo University, Kasr El-Aini Street, Cairo, 11562 Egypt

**Keywords:** Difluprednate, Differential pulse voltammetry, Stability, Greenness and whiteness, Chemistry, Nanoscience and technology

## Abstract

Nowadays, scientists are currently attempting to lessen the harmful effects of chemicals on the environment. Stability testing identifies how a drug’s quality changes over time. The current work suggests a first and sustainable differential pulse voltammetry technique for quantifying difluprednate (DIF) as an anti-inflammatory agent in the presence of its alkaline degradation product (DEG). The optimum conditions for the developed method were investigated with a glassy carbon electrode and a scan rate of 100 mV s^−1^. The linearity range was 2.0 × 10^−7^–1.0 × 10^−6^ M for DIF. DIF was found to undergo alkaline degradation, when refluxed for 8 h using 2.0 M NaOH, and DEG was successfully characterized utilizing IR and MS/MS. The intended approach demonstrated the selectivity for DIF identification in pure, pharmaceutical, and degradation forms. The student’s t-test and F value were used to compare the suggested and reported approaches statistically. The results were validated according to ICH requirements. The greenness of the studied approach was evaluated using the Green Analytical Procedure Index and the Analytical Greenness metric. Additionally, the whiteness features of the proposed approach were examined with the recently released red, green, and blue 12 model, and the recommended strategy performed better than the reported approaches in greenness and whiteness.

## Introduction

**Figure 1:**
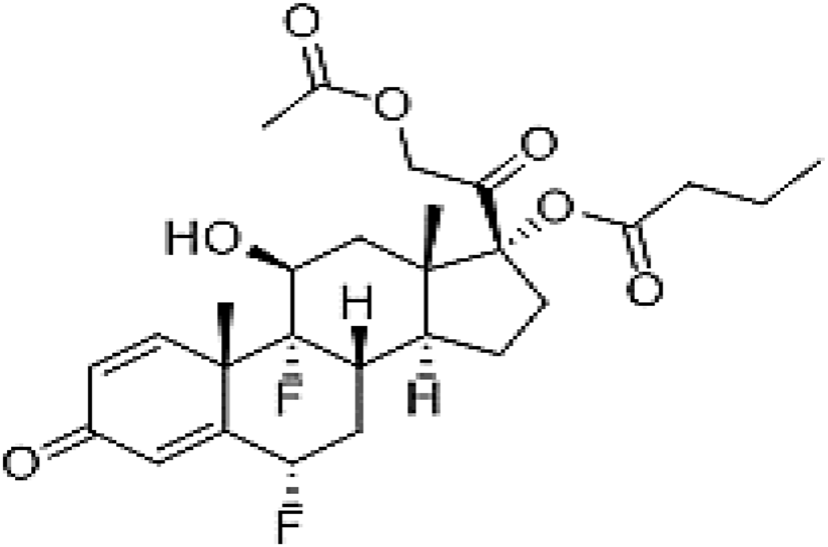
Chemical structure of difluprednate.

Difluprednate (DIF) is a topical corticosteroid used to relieve post-ocular surgery pain and inflammation^1^. Its chemical name is a butyrate ester of (6, 9)-difluoroprednisolone acetate (Fig. 1)^1^. It is reported that corticosteroids function by inducing inhibitory proteins of phospholipase A2. These proteins are considered to prevent the release of arachidonic acid, which is a common precursor to powerful inflammatory mediators like prostaglandins and leukotrienes, by regulating the biosynthesis of these molecules^1^. Diflustero® eye drops, which have DIF as an active ingredient, are prescribed for the treatment of endogenous anterior uveitis.

Green analytical chemistry (GAC) has been popular since the early 2000s. Consequently, researchers are attempting to reduce the hazardous chemicals consumed in conventional analytical techniques to improve analyst and environmental safety^2,3^. Some methods for assessing how ecologically friendly a suggested analytical methodology are the Green Analytical Procedure Index (GAPI) and Analytical GREEnness metric (AGREE)^4^. White Analytical Chemistry (WAC) is an extension of GAC with 12 WAC principles additional principles. WAC takes into account two additional important factors in addition to the green ones that affect the method's quality. These factors are the analytical (red) and practical (blue) aspects^5^. Whiteness can also be measured as a convenient parameter for comparison and method selection, based on the evaluation of individual principles.

Different HPLC approaches for DIF determination in pharmaceutical formulation and aqueous humor were carried out by reviewing the literature^6–10^. Four spectrophotometric methods were reported for the determination of DIF in the presence of its degradation products^11^.  No voltammetric method for determining DIF alone or in the presence of its degradation product has been reported in any publications yet.

To enhance the consistency of the active pharmaceutical ingredient and its pharmaceutical formulation, it is becoming more and more vital to isolate, detect, quantify, and characterize the most likely and potential degradation products. In order to elucidate the inherent stability properties of the active drug, the ICH mandated stress tests as part of the degradation guidelines^12,13^. The best stability-indicating technique is the one that can effortlessly detect and measure the drug's breakdown products^14^.

The study of chemical reactions that result in the movement of electrons is known as electrochemistry. Electrons can move from one element to another in a reaction known as an oxidation–reduction reaction, which produces electricity^15^. Voltammetry is the term for a class of electroanalytical techniques that measure current in an electrochemical cell as a function of applied potential to obtain information about the analyte^16^. A wide range of applications, including basic research on oxidation and reduction processes in diverse media, make use of voltammetric techniques ^17^. Regarding the analysis of various inorganic and organic species, different voltammetric approaches are progressing and receiving a lot of attention. They have inherent benefits like simplicity, high sensitivity, wide linear range, low cost, and quick analysis times. Various types of electrodes, which rely on the electrochemical sensitivity of the analytes towards the electrode, can be employed in these voltammetric methodologies. These include GCE, carbon paste, pencil graphite electrodes and microfabricated electrodes^18^. Among the applicable nanomaterials for GCE modification, GNP have gained a lot of attention. This is remarkably related to their chemical, optical, electrical, and catalytic capabilities^18^. The GNP modification of GCE can be readily accomplished in-situ, from chloroauric acid solution, using electrochemical techniques, such as cyclic voltammetric and chronoamperometric methods^19^. The nanoparticles size could be controlled by choosing the optimum experimental parameters, such as cycles number, scan rate, and potential range.

The objective of this study is to evaluate the performance of a novel, selective, and eco-friendly differential pulse voltammetric (DPV) method for determining DIF in the presence of its alkaline degradation product (DEG). Different experimental parameters, including the electrode and scan rate, were examined and optimized. The established method was successful in estimating DIF in both pure form and pharmaceutical dosage form. The developed technique was compared to the published HPLC method in terms of green assessment using GAPI and AGREE in addition to whiteness assessment using the red, green, and blue (RGB12) algorithms. Finally, the suggested method was statistically compared to the reported HPLC method^7^.

## Materials and methods

### Chemicals, reagents, and pharmaceutical formulation

Orchidia Co. for Pharmaceutical Ind. (Cairo, Egypt) graciously provided DIF used in this study. The official method revealed that its purity was 98.05% ± 0.50 for DIF^20^. Tetrabutylammonium tetrafluoroborate (TBATFB) used as a supporting electrolyte and was supplied by Sigma-Aldrich. Ethanol and NaOH from El-Nasr Pharmaceutical Chemicals Co. (Cairo, Egypt) and HAuCl_4_ (Sigma-Aldrich) (Cairo, Egypt) were used. Diflustero® eye drop (Orchidia. Co for Pharmaceutical Ind, Cairo, Egypt), (Batch No. (10) 1220140), claimed to have 0.5 mg of DIF per mL, was purchased from the local market**.**

### Preparation of standard solutions

Tetrabutylammonium tetrafluoroborate solution (0.01 M) was prepared by dissolving 3.293 gm in a 1000-mL measuring flask and completing the volume with ethanol. The previous solution was used as a solvent in the studied voltammetric method.

Stock standard solution of DIF was prepared by weighing and accurately transferring 0.0127 gm of pure drug into 25-mL measuring flask. The volume was completed with 0.01 M of TBATFB solution to reach final concentration of 1.0 × 10^–3^ M.

### Preparation of degradation product

To 100 mg of DIF, 10 mL ethanol and 50 mL 2.0 M NaOH were added and refluxed at 120 °C for 8 h. After neutralization with 2.0 M HCl, the previous solution was poured into a 100-mL measuring flask and filled to its capacity with ethanol. The solution was then evaporated in beaker until it was completely dried and then dissolved in ethanol. Using the previously mentioned electrolyte (DIF), the degraded solution was further diluted to yield a concentration of 4.0 × 10^–7^ M, which was utilized in DPV, Fourier-transform infrared (FT-IR) spectroscopy and mass spectrometry.

### Apparatus

A PC-controlled electrochemical analytical workstation (Metrohm Autolab potentiostat/galvanostat PGSTAT204), equipped with NOVA 1.11.1 software for electrochemistry, was used to perform all voltammetric measurements. In established voltammetric method, three electrodes are used: a reference Ag/AgCl electrode (3 M KCl), glassy carbone electrode (GCE) as a working electrode and an auxiliary or counter electrode Pt wire.

### Optimization of experimental conditions and modification of electrode

Different electrodes were tried, such as pencil graphite electrodes and GCE. In addition, several scan rates were examined at 10, 25, 50, 75, 100 and 120 mV s^1^ to measure 4.0 × 10^–7^ of DIF in the presence of its alkaline degradation product. As previously described in the literature^18^, the GCE was modified by gold nanoparticles (GNP) in a 0.1 M KCl supporting solution containing 0.5 mM HAuCl_4_ using cyclic voltammetry (2.0 to - 2.0 V scanning potential range, 100 mV s^−1^ scan rate, and 8.0 mV step potential for 20 consecutive cycles).

### Method validation

The proposed DPV voltammetric technique has been validated in compliance with ICH guidelines^13^. Appropriate aliquots of DIF were carefully transferred from stock solution to 25-mL measuring flasks to reach the five concentrations of DIF using the optimized voltammetric technique. DPV was recorded over a range of -1.2 to - 2.0 V. Each sample's peak current height (*ip*) was measured, and the calibration curve was made by plotting each value against the matching concentration. Using the recommended DPV technique, three DIF concentrations were tested for accuracy within its linearity range. The concentrations were then calculated using the corresponding regression equation. The previously described procedure was used in triplicates intra-daily to analyze three concentrations of under linearity. The relative standard deviation (RSD%) for each sample was then estimated to check repeatability. Using the method specified under linearity, the aforementioned DIF samples under repeatability were examined in triplicate over the course of three days to assess intermediate precision. After then, each sample's RSD% was determined. Limit of detection (LOD) and limit of quantification (LOQ) were calculated from the standard deviation (s) of the response and the slope of the calibration curve (S) according to the following equations: LOD = 3.3 (s/S) and LOQ = 10 (s/S).

### Application to the pharmaceutical formulation and statistical analysis study

A precise transfer of 0.4 mL of Diflustero® was applied into a 25-mL measuring flask, and the volume was adjusted with 0.01 M of TBATFB solution to reach a concentration of 1.0 × 10^–3^. Using the previously optimized parameters within the calibration curve construction process, DPV was used to prepare and measure three distinct concentrations that fell within the linearity range of DIF. The outcomes of the applied method were compared to those of the reported HPLC method as well as to one another using student t-test and F value^7^.

## Result and discussion

Therefore, this work's objective was to examine and enhance the experimental setup to create a smart, white, and green DPV method that indicates stability and can be described to determine DIF in the presence of DEG. GCE has many outstanding properties that make it useful in electrocatalytic applications. These qualities include low cost, excellent electrical conductivity, electrochemical inertness over a broad potential window, high hardness, chemical stability, impermeability, and ease of surface modification^21^.

### Electrochemical behavior of DIF

**Figure 2:**
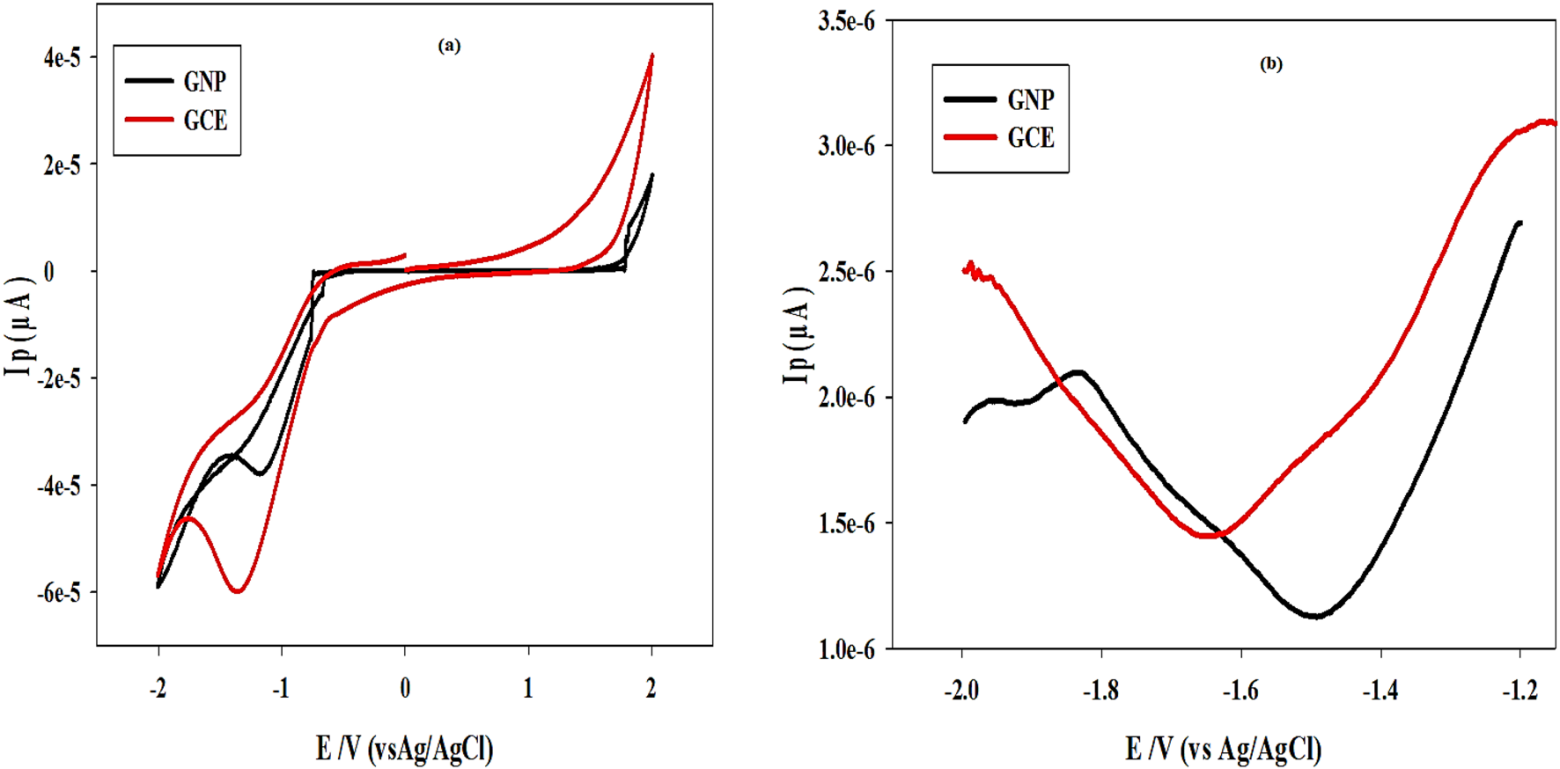
(**a**) Cyclic voltammograms of 4.0 × 10^−7^ M difluprednate using glassy carbon electrode and gold nanoparticle electrode recorded versus Ag/AgCl reference electrode. (**b**) differential pulse voltammograms of 4.0 × 10^−7^ M difluprednate at glassy carbon electrode and gold nanoparticle electrode.

A redox probe that is sensitive to the state of the carbon surface in 0.1 M KCl solution, 10.0 mM K_3_Fe (CN)_6/_K_4_Fe (CN)_6_, was used to perform DPV characterization of bare GCE. The cyclic voltammogram (CV) in Fig. 2a, clearly illustrates the results, which indicates that the current peak height (*i*_*p*_) is negatively impacted using the GCE modified with GNP. As of right now, the peak height (i_p_) of DIF for bare GCE was 5.99 × 10^–5^ μA, while for GCE modified with GNP, it was 3.47 × 10^–5^ μA.

The electrochemical reduction of DIF was examined using bare GCE and GCE modified with GNP applied against a Ag/AgCl reference electrode between  -1.2 and - 2.0 V. Figure 2b displays DPV voltammogram of DIF at the bare GCE and GCE modified with GNP surface in order to examine the electrocatalytic activity of GCE and its possible application for augmenting the current peak of DIF. The electrocatalytic behavior, the facilitated electron transfer rate at the GCE surface, and the increase in electrode surface area are all responsible for the higher anodic current sensitivity seen in Fig. 2b, when compared to GCE modified with GNP. So, bare GCE was chosen as a working electrode for the proposed voltammetric method due to modification with GNP didn’t enhance the peak current of DIF.

### Effect of scan rate

**Figure 3:**
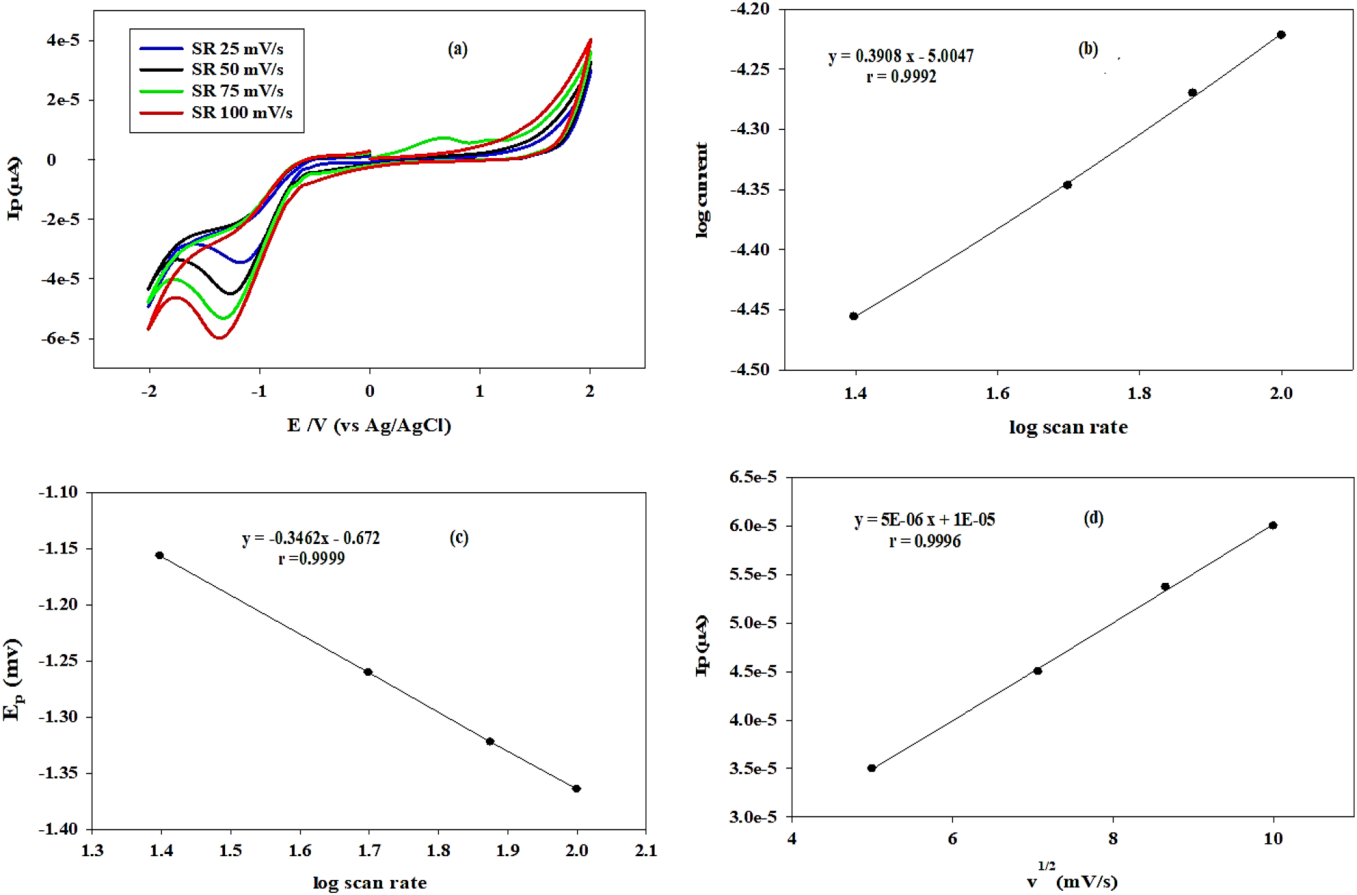
(**a**) Cyclic voltammograms of 4.0 × 10^−7^ M difluprednate at glassy carbon electrode as a function of scan rate (25–100 mV s^−1^). (**b**) The relation between log anodic peak current of difluprednate and log scan rate at glassy carbon electrode. (**c**) The relation between peak potential of difluprednate and log scan rate at glassy carbon electrode. (**d**) The relation between peak current of difluprednate and the square root of scan rate at glassy carbon electrode.

Using DPV over the range of 25–100 mV s^−1^, the effect of scan rate on the anodic reduction peak of 4.0 × 10^−7^ M of DIF was investigated, as illustrated in Fig. 3a. The oxidation peak in Fig. 3a may represent the oxidation of the hydroxyl group to the carbonyl group. Peak potential (E_p_) was clearly shifted to a more negative potential, confirming that E_p_ is dependent on scan rate and that the reduction process is irreversible. Plotting log current versus log scan rate (υ) yields a straight line using the following regression equation as noticed in Fig. 3b, log *i* = 0.3908 log υ-5.0047 (r = 0.9992). The subsequent equation can be utilized to express the relationship between the log scan rate and the E_P_ of DIF: E_*p*_ =- 0.3462 υ^1/2^ to 0.672 (r = 0.9999) as shown in Fig. 3c. The slope is less than 0.50, indicating that diffusion is likely controlling the reaction occurring at the electrode's surface. Additionally, by increasing the scan rate from 25 to 100 mV s^−1^, as illustrated in Fig. 3d, a direct proportionality was found between the (E_p_) and the square root of the scan rate (υ^1/2^), according to the equation E_p_ = 5E-06 υ^1/2^ + 1E-05 (r = 0.9996).

### Degradation elucidation of DIF

**Figure 4:**
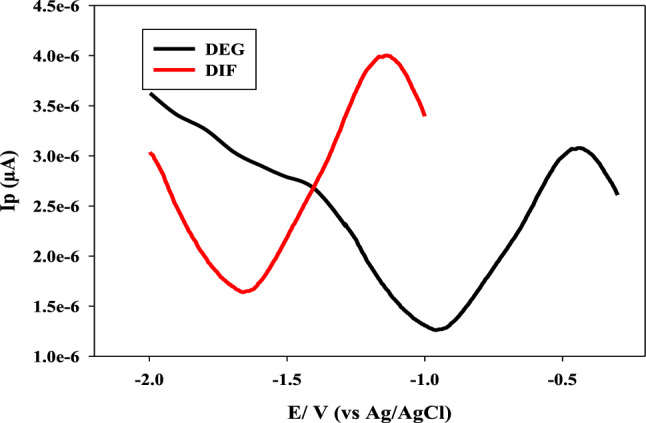
Differential pulse voltammograms of 4.0 × 10^−7^ M difluprednate and its alkaline degradation product at glassy carbon electrode.

Firstly, we tried various degradation conditions, according to ICH guidelines like acid, alkaline, oxidation, and sunlight, but DIF remained stable under the previous conditions except alkaline condition. During trials with gradually increasing NaOH molarities of 0.5, 1.0, 1.5, and 2.0 M, it was discovered that every molarity resulted in the same degradation pattern. By using 2.0 M NaOH, the hydrolysis of DIF was completed faster. After refluxing with 2.0 M NaOH for 8 h., DIF was degraded, (Fig. 1S). The DEG assignment was determined using the degradation products' FT-IR and mass spectral data. The ester group that corresponds to the suggested DEG at 1730 cm^−1^ was not present in the FT-IR spectrum, (Fig. 2S)^22^. The intact DIF's calculated molecular weight is 505.5 m/z^20^, and the DEG's structure was validated by MS analysis with a molecular weight of 338.15 m/z, (Fig. 3S). There was no interference between DIF and DEG’s DPV voltammograms which is shown in Fig. 4.

### Reduction mechanism of DIF

Due to the ketone group in DIF being reduced to a hydroxyl group, two electrons were gained during the reduction process^22^. Figure 4S illustrated how DIF is reduced at the selected electrode.

### Method validation

**Figure 5:**
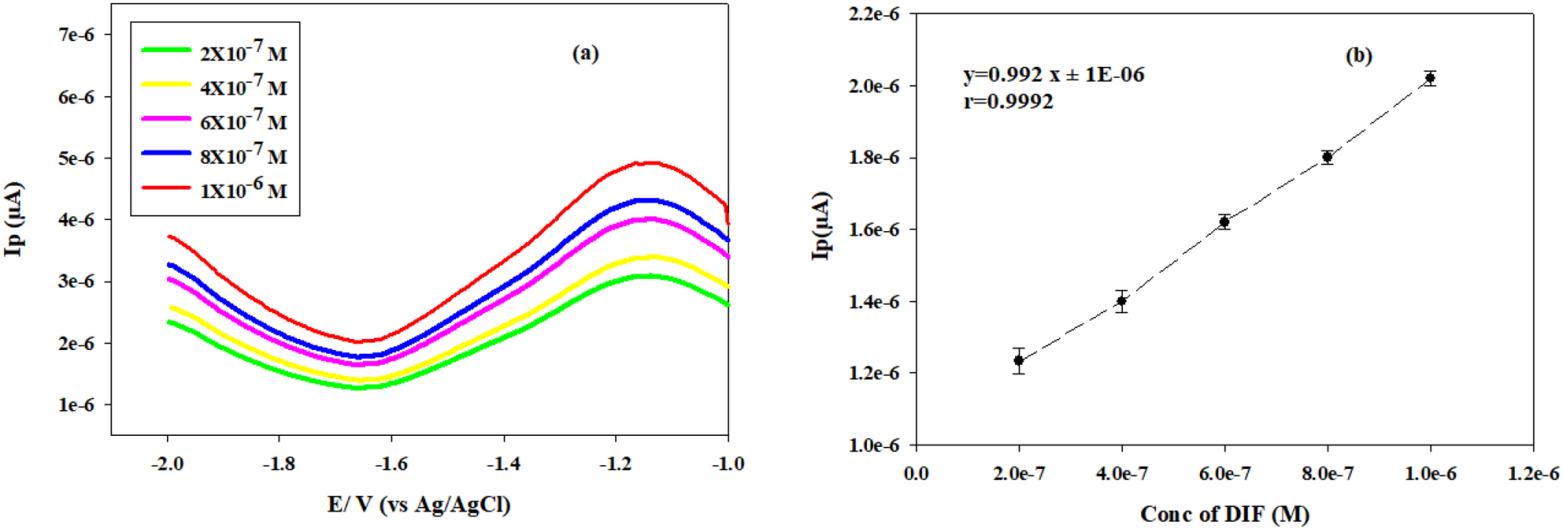
(**a**) Differential pulse voltammograms of concentration range (2.0 × 10^−7^–1.0 × 10^−6^ M) of difluprednate solution at glassy carbon electrode. (**b**) The calibration curve of differential pulse voltammograms of different concentrations of difluprednate solution with error bars at glassy carbon electrode.

Under optimal electrochemical conditions, the DPV method was found to be linear within the range of 2.0 × 10^–7^–1.0 × 10^–6^ M, Fig. 5a. The regression equation for the method was as follows: *Ip* (μA) = 1E-06 + 0.0992 c, r = 0.9992, as illustrated in Fig. 5b. Table 1 presents a summary of the results of the calculation of various validation parameters in accordance with ICH guidelines.

**Table 1:** Validation parameters of the proposed voltammetric method for determination of pure samples of difluprednate.

Parameters	DIF
Linearity range (M)	2.0 × 10^–7^–1.0 × 10^–6^
Correlation coefficient (r)	0.9992
Slope	0.992
Intercept	1.0 × 10^–6^
Accuracy^a^ (mean ± SD)	100.38 ± 1.11
Precision^b^ (RSD %)	
Repeatability	0.79
Intermediate precision	1.18
LOD^c^ (M)	4.98E−08
LOQ^c^ (M)	1.49E−07

^a^n = 9, ^b^n = 9. ^c^LOD and LOQ were calculated from the standard deviation (s) of the response and the slope of the calibration curve (S) according to the following equations: LOD = 3.3 (s/S) and LOQ = 10 (s/S).

The accuracy was ascertained by computing the recovery percentage for each of the three replicates at three distinct concentrations (3.0 × 10^–7^, 5.0 × 10^–7^, and 7.0 × 10^–7^ M) that confirm the DIF's linearity range as indicated in Table 1. The recovery % and SD values were equal to 100.38% and 1.11, respectively. Repeatability and intermediate precision were determined as the RSD% and were found to be less than 2% as demonstrated in Table 1, indicating that the technique adopted was sufficiently precise to be used during routine work. The values of concentrations that used in precision were the same applied in accuracy. RSD% values of repeatability and intermediate precision were 0.79 and 1.18, respectively. Values of LOD and LOQ were 4.98E−08 and 1.49E−07, respectively as shown in Table 1.

### Application to the pharmaceutical formulation and statistical analysis study

Applying the proposed methodology, Table 2 displays DIF determination in its pharmaceutical product. When compared to the published HPLC method, the suggested method was statistically tested and found to have no significant difference, demonstrating that the applied method is accurate and precise Table 3.

**Table 2:** Determination of difluprednate in a pharmaceutical formulation using the proposed sensing protocol.

Sample	Amount added standard (M)	Apparent recovery %
Diflustero ® eye drop (0.5 mg of DIF per 1.0 mL)	4.0 × 10^–7^	98.63
	6.0 × 10^–7^	99.75
	8.0 × 10^–7^	99.63
Recovery% ± SD		99.34 ± 0.61

**Table 3:** Statistical comparison of the obtained results by applying the proposed voltammetric method and the reported method for determination of diflupredenate in Diflustero® eye drop.

Value	Proposed DPV method	Reported method^a^
Mean (%)^b^	99.34	101.00
SD	0.61	0.57
n	6	6
Variance	0.37	0.32
Student’s t-test (2.23)^c^	0.21	–
F value (5.05)^c^	1.15	–

^a^HPLC method using phosphate buffer (pH 6) and acetonitrile (50:50, v/v) at a flow rate of 1.2 mL min^−1^, detection at 240.0 nm^7^. ^b^Average of six experiments. ^c^Figures between parentheses represent the corresponding tabulated values of* t* and F- at *P* = 0.05.

### Greenness assessment

The ecological consequence of the analytical techniques was assessed using four main criteria: high energy consumption, high waste production, excessive chemical use and associated risks, and large chemical usage^23,24^. The cost-effectiveness and environmental friendliness of the proposed method were assessed using two analytical techniques as follows:

#### Green analytical procedure index (GAPI)

**Figure 6:**
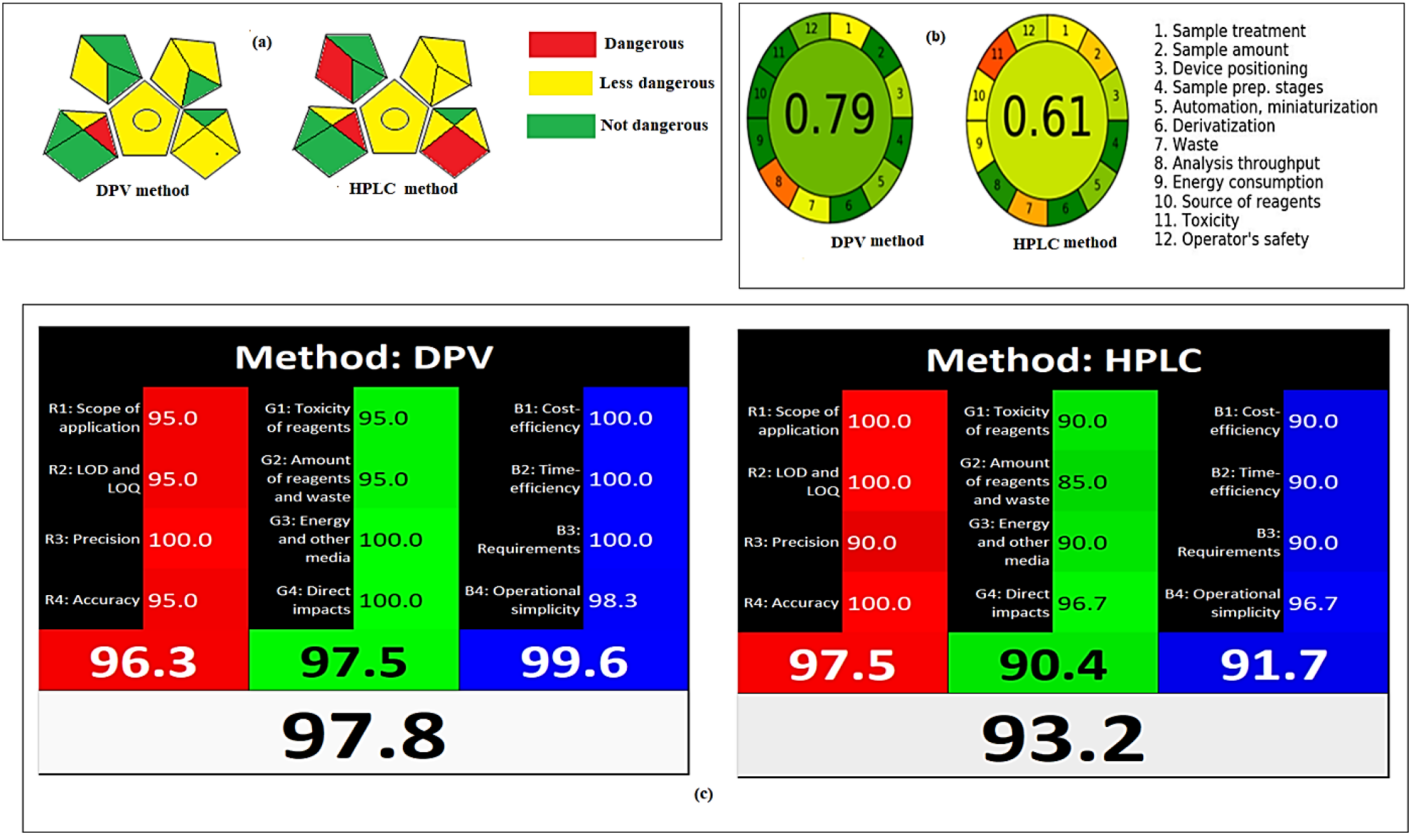
(**a**) GAPI pictograms for suggested and reported methods. (**b**) AGREE assessment of the green profile for suggested and reported methods. (**c**) Whiteness assessment of the suggested and reported approaches by the RGB12 algorithm.

This tool provides an in-depth understanding of fifteen analytical technique areas, each of which is represented by five pentagrams and represents a step in the analysis methodology, such as sampling, preparation, instrumentation, solvents, and reagents used, and the goal of the analytical method. The GAPI color scheme states that green indicates a higher ecological tolerance, yellow refers to a lesser ecological tolerance, and red reveals a significant environmental risk^25,26^. The GAPI tool evaluated analytical procedures and provided them with a green assessment profile, as shown in Fig. 6a. The proposed method has six green and one red pictograms, while the reported method has five green and three red pictograms. So according to the results from Fig. 6a, the established DPV method was greener than the reported HPLC method.

#### Analytical GREEnness metric (AGREE)

The 12 principles of GAC, which include treatment, sample amount and stage, waste, energy consumption, and toxicity, are used as assessment criteria^27–29^. A single 0–1 scale is created from each of these variables. In the central region of the pictogram, the evaluated methods are classified as being more environmentally friendly, if values are close to 1 with a dark green background^2^. These variables and a comparison of the suggested and reported methods are displayed as colored pictograms in Fig. 6b. The established method had an AGREE score of 0.79, compared to 0.61 for the reported method. The developed approach is therefore more environmentally friendly than the reported HPLC approach, as determined by the AGREE score.

### Whiteness assessment

The 12 WAC principles are divided into 4 red principles, 4 green principles, and 4 blue principles^5^. The red ones represent the analytical performance (scope of application, LOD and LOQ, precision and accuracy), the green ones represent to the green chemistry (toxicity of reagents, number and amount of reagents and waste, energy and other media and direct impacts), while the blue ones represent the practical side (cost-efficiency, time-efficiency, requirements and operational simplicity)^5^. The whiteness scores for the suggested and reported approaches were calculated. Zero score is for the worst result while 100 score means that the method is well fitted for a planned application with respect to principle. An analysis of whiteness using the RGB12 model which states that the combination of red, green, and blue light beams creates the illusion of whiteness that reveals the coherence and synergy of the analytical, ecological, and practical features^30^.

Red, green, and blue principles which stand for whiteness scores were computed for the suggested and reported methods. These results are shown in Fig. 6c, which reveals the superiority of the suggested approach from the standpoint of whiteness as the suggested voltammetric method has score 97.8 while the reported HPLC method has score 93.2.

## Conclusion

Voltammetric techniques are widely used in many different contexts, such as basic studies of oxidation and reduction processes in a range of media. The DPV approach utilized GCE to show the first electrochemical determination of DIF in the presence of its alkaline degradation product. Different conditions of degradation were tried but alkaline degradation showed the most degradable product. In addition to being an excellent green and white analytical method, this method was applied with many advantages over other reported methods, including short analysis times, straightforward procedures with good validation parameters, and affordable instrumentation. The recommended voltammetric method worked well for figuring out DIF in both bulk form and dosage formulation as well as in the presence of its alkaline degradation product. The proposed method outperformed the published approaches when evaluated for greenness using two greenness assessment metrics (GAPI and AGREE) and for whiteness using the RGB12 algorithm.

### Supplementary Information

Supplementary Figures.

## Data Availability

Data is provided within the manuscript or supplementary information files.
