# Supplementary material for: Greenness and whiteness assessment of a sustainable voltammetric method for difluprednate estimation in the presence of its alkaline degradation product
Source: Sci Rep. 2024 May 27;14:12088. doi: 10.1038/s41598-024-61712-0 (PMC11130124; doi:10.1038/s41598-024-61712-0)
Supplement: Supplementary file 1 — Supplementary Figures. [file 41598_2024_61712_MOESM1_ESM.pdf]

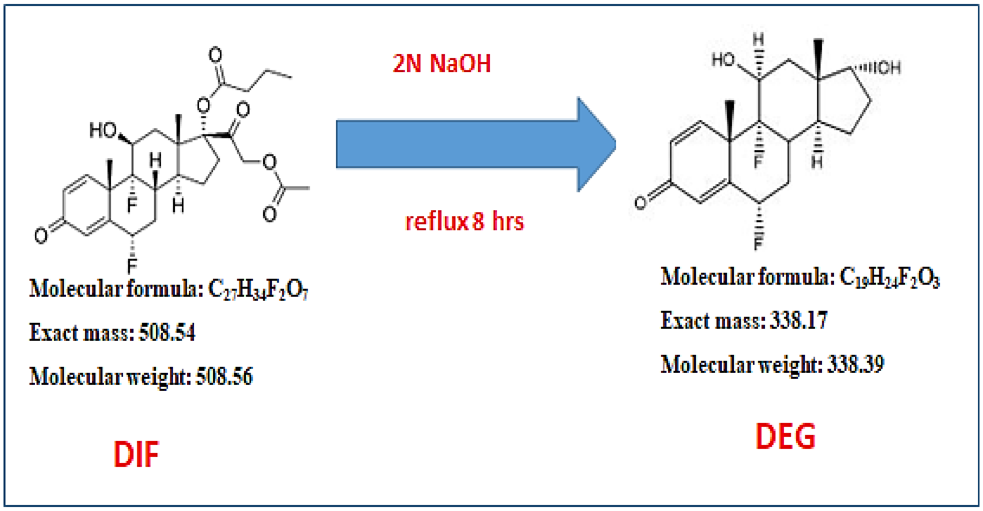

Fig. 1S: Schematic diagram showing the suggested degradation pathway of difluprednate.

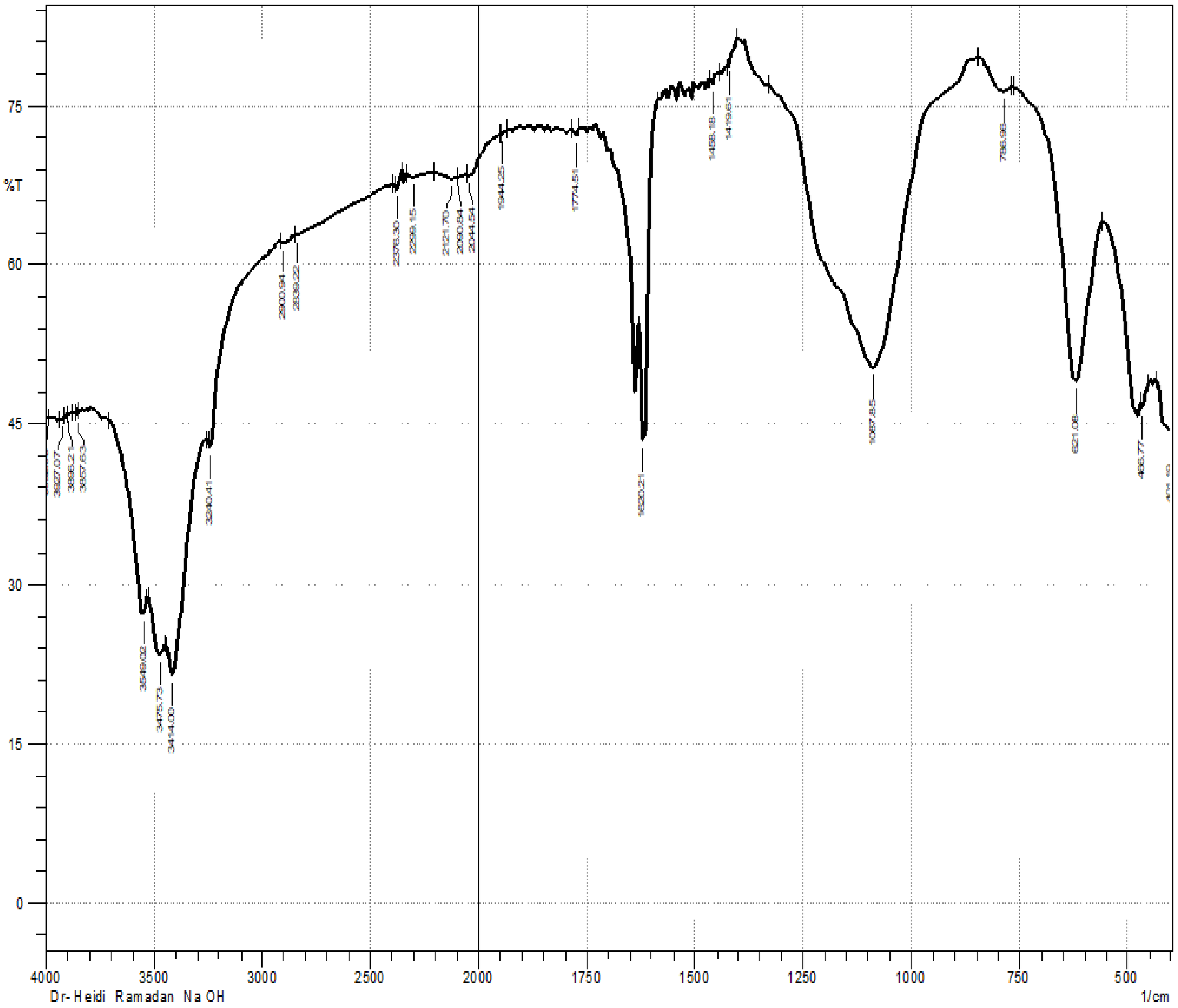

Fig. 2S: FT- IR spectrum of alkaline-induced degradation product of difluprednate.

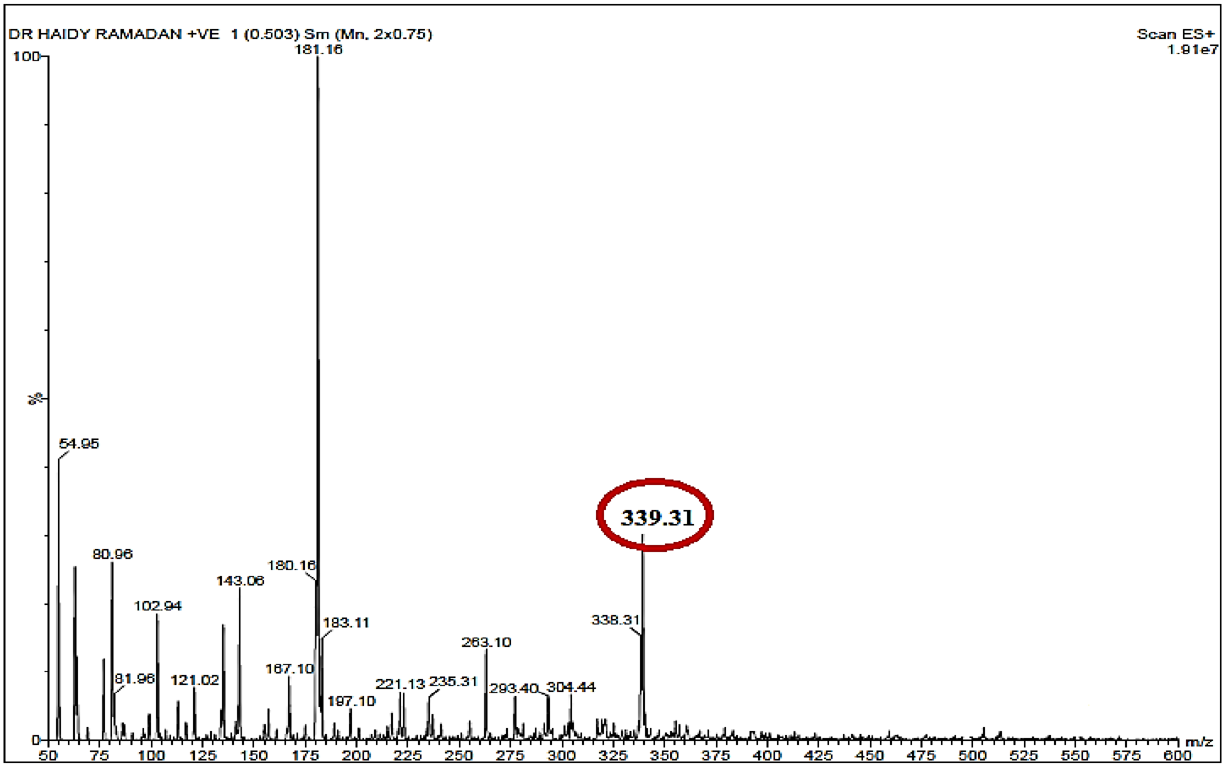

Fig. 3S: MS/MS spectrum of alkaline-induced degradation product of difluprednate.

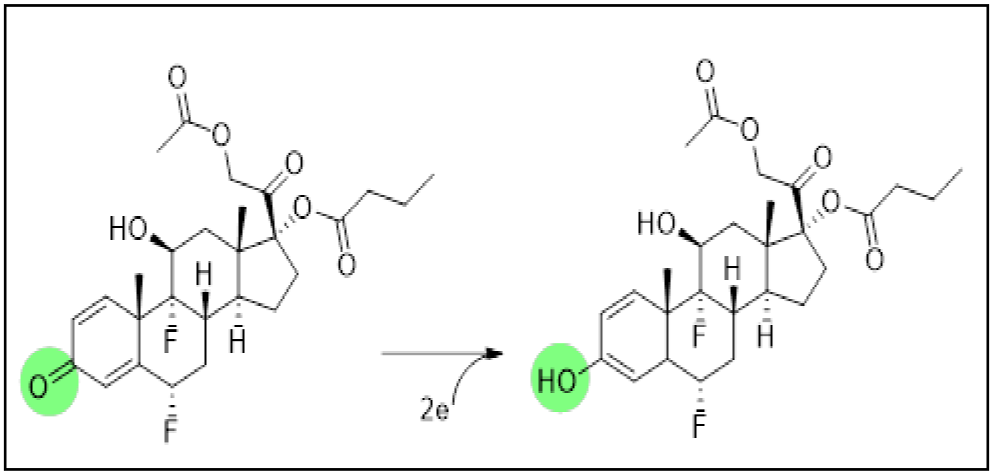

Fig. 4S: Proposed electrochemical reduction reaction of difluprednate.
